# Factors associated with quality of life in patients with central centrifugal cicatricial alopecia: A cross-sectional study

**DOI:** 10.1016/j.jdin.2024.02.004

**Published:** 2024-02-16

**Authors:** Li-Chi Chen, Chino Ogbutor, Ogechi Ezemma, Shivali Devjani, Kristen J. Kelley, Maryanne M. Senna

**Affiliations:** a Department of Dermatology, Lahey Hospital & Medical Center, Burlington, Massachusetts; b Department of Dermatology, Harvard Medical School, Boston, Massachusetts

**Keywords:** alopecia, CCCA-QLI, central centrifugal cicatricial alopecia, hair loss, quality of life, scarring alopecia

*To the Editor:* Central centrifugal cicatricial alopecia (CCCA) is a primary scarring alopecia with an incidence of 5.6% in African-American women in the United States.^1^ Despite an accumulating amount of literature documenting psychological distress and impaired quality of life (QoL) in individuals with scarring alopecia,^2^ there is a paucity of studies exploring the factors related to QoL in patients with CCCA specifically. In this study, we sought to determine the sociodemographic and clinical variables associated with QoL in patients with CCCA.

A survey with the Central Centrifugal Cicatricial Alopecia Quality of Life Index (CCCA-QLI) questionnaire was administered on Scarring Alopecia Foundation social media platforms. The CCCA-QLI comprises 21 items to be rated on a 4-point scale, with responses ranging from "not affected at all (1)" to "highly affected (4)." Scores are calculated as the sum of the responses, and higher scores indicate worse QoL. Purposeful selection of covariates was conducted using the univariate screening (Table I). Factors associated with CCCA-QLI scores at a significance level <0.15 and confounders, defined as variables that changed the estimate of another variable by >15% when separately added to the model, were incorporated into the final model.^3^ Multivariate linear regression was performed to evaluate the adjusted associations between the selected factors and CCCA-QLI scores.

**Table I:** Patient characteristics of the study population and the mean Central Centrifugal Cicatricial Alopecia Quality of Life Index scores (*N* = 97)

Characteristics	*n* (%)	Mean CCCA-QLI score (SD)	*P* value
Gender			—
Female	97 (100)	55.7 (12.6)	
Age			.149
<40 y	17 (17.5)	59.7 (11.6)	
41-65 y	64 (66.0)	55.3 (13.3)	
>65 y	16 (16.5)	53.8 (11.5)	
Race/ethnicity			.946
African American	89 (91.8)	55.8 (12.8)	
White	4 (4.1)	55.0 (14.7)	
Others	4 (4.1)	53.8 (11.5)	
Education level (*n* = 95)∗			.006
High school or less	23 (24.2)	61.4 (9.77)	
College degree	24 (25.3)	57.8 (13.0)	
Graduate degree	48 (50.5)	52.1 (11.9)	
Occupational status (*n* = 95)∗			.814
Active	67 (70.5)	55.9 (12.8)	
Inactive (retired, unemployed)	28 (29.5)	56.6 (11.6)	
Disease duration			.109
<5 y	42 (43.3)	55.1 (11.4)	
5-10 y	23 (23.7)	52.0 (14.6)	
>10 y	32 (33.0)	59.1 (12.2)	
Self-reported CCCA severity			.037
1	15 (15.5)	46.6 (12.0)	
2	17 (17.5)	56.1 (12.3)	
3	18 (18.6)	55.8 (10.5)	
4	24 (24.7)	58.8 (12.7)	
5	23 (23.7)	58.0 (13.0)	
Self-reported CCCA subtype			.971
A (frontal accentuation)	54 (55.7)	55.6 (12.1)	
B (vertex accentuation)	43 (44.3)	55.7 (13.5)	
Family history			.785
No	45 (46.4)	55.3 (12.8)	
Yes	52 (53.6)	56.0 (12.6)	
Frequency of wearing a wig			<.001
Never/almost never	59 (60.8)	52.3 (11.9)	
Always/almost always	38 (39.2)	60.9 (12.1)	

*CCCA*, Central centrifugal cicatricial alopecia; *CCCA-QLI*, Central Centrifugal Cicatricial Alopecia Quality of Life Index.

∗ Sample sizes may vary because of unreported data, and percentages are calculated based on the complete data of the column.

A total of 97 individuals with self-reported CCCA (84.5% with self-reported biopsy-proven CCCA) initiated the survey, with 97 surveys completed in full (completion rate: 100%). The mean age of the participants was 52.5 years (SD, 11.7), the mean disease duration was 7.8 years (SD, 7.7), and the mean self-reported severity was 3.2 (SD, 1.4). Younger age (*P* = .030) and longer disease duration (*P* = .026) were associated with significantly higher CCCA-QLI scores, indicating worse QoL than patients with CCCA who were older and had a shorter disease duration. Additionally, patients with frequent wig use reported worse QoL (*P* = .015) than those who never or rarely wore a wig. Furthermore, a graduate-level education was correlated with improved QoL compared to a high school education or less (*P* = .002) (Table II).

**Table II:** Multivariate linear regression model of factors associated with Central Centrifugal Cicatricial Alopecia Quality of Life Index scores (*N* = 97)

Characteristics	Adjusted coefficient	*P* value	95% CI	
Age∗	−0.30	**.030**	−0.56	−0.03
Education level				
High school or less	Reference	—	—	—
College degree	−6.02	.078	−12.73	0.69
Graduate degree	−9.07	**.002**	−14.79	−3.35
Occupational status				
Active	Reference	—	—	—
Inactive (retired, unemployed)	4.82	.149	−1.77	11.41
Disease duration∗	0.39	**.026**	0.01	0.69
Self-reported CCCA severity				
1	Reference	—	—	—
2	7.88	.056	−0.22	15.98
3	5.78	.154	−2.21	13.77
4	5.81	.147	−2.09	13.72
5	1.67	.696	−6.82	10.16
Frequency of wearing a wig				
Never/almost never	Reference	—	—	—
Always/almost always	6.75	**.015**	1.11	11.9

Bold *P*-values are statistically significant (*P* < .05).

*CCCA*, Central centrifugal cicatricial alopecia.

∗ Age and disease duration are treated as continuous variables, and the adjusted coefficients and CIs presented are changes of Central Centrifugal Cicatricial Alopecia Quality of Life Index scores with a 1-year increase in age or disease duration.

In accordance with our findings, a previous study identified younger age and longer disease duration as predictors for worse QoL in alopecia areata, while QoL was not affected by a family history of alopecia areata.^4^ In a survey study of 338 participants with alopecia, 49% reported wearing a wig had a negative impact on everyday life due to concerns about others knowing it was a wig, the wig falling off, and discomfort, which led to avoidance of social situations and exercise.^5^ Although educational disparities in QoL are yet to be documented in the context of alopecia, our findings might offer insights into the positive relationship between education level and personal psychosocial resources critical for coping with adversities during the course of hair loss.

Our study is limited by potential misclassification of self-reported CCCA diagnosis/severity and self-selection bias due to social media recruitment but adds important information to the literature in identifying the determinants for QoL in CCCA. Dermatologists should consider sociodemographic and clinical factors during patient counseling and treatment plan development to enhance patient well-being, especially among younger patients, those with longer disease duration, fewer formal years of education, and frequent wig use.

## Conflicts of interest

None disclosed.
